# Peripheral immunity patterns, imaging features, and clinical outcomes in patients with gait impairment and ventriculomegaly on brain MRI

**DOI:** 10.3389/fnagi.2025.1685288

**Published:** 2025-10-20

**Authors:** Christian Espinoza-Vinces, Iciar Aviles-Olmos, Jorge M. Núñez-Córdoba, Marcos Jiménez-Vázquez, Marta Calvo-Imirizaldu, Genoveva Montoya-Murillo, Gloria Martí-Andrés, Javier Arbizu, María-Rosario Luquin

**Affiliations:** ^1^Department of Neurology, Clínica Universidad de Navarra, Pamplona, Spain; ^2^Biomedical Engineering Program/Innovative Therapies Division, Center for Applied Medical Research, Pamplona, Spain; ^3^Navarra Institute for Health Research (IdiSNA), Pamplona, Spain; ^4^Research Support Service-Central Clinical Trials Unit, Clínica Universidad de Navarra, Pamplona, Spain; ^5^Department of Radiology, Clínica Universidad de Navarra, Pamplona, Spain; ^6^Physiological Monitoring and Control Laboratory, Biomedical Engineering Program, Center for Applied Medical Research (CIMA), Pamplona, Spain; ^7^Department of Nuclear Medicine, Clínica Universidad de Navarra, Pamplona, Spain

**Keywords:** idiopathic normal pressure hydrocephalus, neutrophil-to-lymphocyte ratio, neurodegeneration, Evans’ index, callosal angle, disproportionately enlarged subarachnoid space hydrocephalus (DESH)

## Abstract

**Introduction:**

Ventricular enlargement is a common finding on brain MRI in patients with gait impairment, particularly in those with idiopathic normal pressure hydrocephalus (iNPH). However, iNPH shares several clinical and radiological features with neurodegenerative diseases, which complicates accurate diagnosis. This study aimed to explore the associations between peripheral immune markers, imaging biomarkers, and final diagnosis in patients with gait disturbance and ventriculomegaly.

**Methods:**

We retrospectively analyzed 55 patients with gait impairment and ventriculomegaly, and 40 age-comparable healthy controls. Clinical assessments included the iNPH Grading Scale (iNPHGS) and cognitive tests. The neutrophil-to-lymphocyte ratio (NLR) was calculated as a peripheral immune marker. Imaging biomarkers included Evans’ index (EI), callosal angle (CA), and disproportionately enlarged subarachnoid space hydrocephalus (DESH) score. Additional cerebrospinal fluid biomarkers and [^18^F]-fluorodopa PET/CT were used when clinically indicated. Patients were classified into two groups at the 5-year follow-up based on current clinical diagnostic criteria, integrating longitudinal clinical evaluation and ancillary investigations. The first group consisted of 35 patients (64%) with a neurodegenerative disorder (ND group), of whom 24 (69%) met criteria for progressive supranuclear palsy, 8 (23%) for Alzheimer’s disease, 2 (6%) for Lewy body dementia, and 1 (3%) for Parkinson’s disease. The remaining 20 patients (36%) fulfilled criteria for probable iNPH and were classified into the iNPH group.

**Results:**

ND patients had significantly higher NLR (*M* = 2.4, SD = 0.5) than iNPH patients (*M* = 1.9, SD = 0.4) and controls (*M* = 1.6, SD = 0.2; *p* < 0.001). NLR distinguished ND from iNPH with an AUC of 0.79 (80% sensitivity, 70% specificity at a cutoff of 2.0). CA demonstrated strong discrimination (95% sensitivity, 86% specificity). Compared to iNPH, ND had higher iNPHGS scores, greater DESH, and lower CA. Baseline NLR correlated with iNPHGS in ND patients (rs = 0.48, *p* = 0.004) but not in iNPH. NLR predicted tap test response differently; higher NLR was linked to non-response in ND, and lower NLR associated with improvement in iNPH.

**Conclusion:**

NLR may serve as a promising peripheral biomarker to differentiate ND from iNPH in patients with gait impairment and ventriculomegaly. Integrating immune, clinical, and imaging markers could improve diagnostic accuracy and guide appropriate therapeutic strategies.

## Introduction

Ventriculomegaly is a common finding on brain MRI in older adults, with a prevalence ranging from 10.2 to 22 per 100,000 (Andersson et al., 2019). Some of these patients exhibit gait disturbances as a principal clinical manifestation and are frequently diagnosed with idiopathic normal pressure hydrocephalus (iNPH). However, iNPH is a complex syndrome with heterogeneous manifestations, including motor and gait disturbances, cognitive decline, and sphincter incontinence, along with ventricular enlargement as the main imaging feature (Ghosh and Lippa, 2014).

Emerging evidence indicates that iNPH may be associated with an increased risk of developing neurodegenerative diseases such as Alzheimer’s disease (AD), Lewy body dementia (LBD), progressive supranuclear palsy (PSP), and multiple system atrophy (MSA) (Espay et al., 2017). In fact, differentiating true iNPH from neurodegenerative diseases with prominent ventricular enlargement can be extremely challenging. Therefore, there is an urgent need to find biomarkers to assist in the differential diagnosis of patients presenting with ventricular enlargement and motor symptoms such as gait disturbances. Potential biomarkers for a correct diagnosis include *β*-amyloid 42, total tau (t-tau), and the neutrophil-to-lymphocyte ratio (NLR) (Espay et al., 2017).

Peripheral immune markers, particularly the NLR, have gained increasing attention as indicators of systemic inflammation in neurodegenerative conditions. Importantly, although reference ranges for NLR exist in healthy populations, no universally accepted cutoff is available, and values can vary across laboratories and populations. The majority of studies have therefore compared mean values in patients with neurodegenerative disorders to those of healthy, age-matched controls, typically using automated hematology analyzers (Bissacco et al., 2023; Mohammadi et al., 2024; Muñoz-Delgado et al., 2024). Previous studies have reported higher NLR values in patients with neurodegenerative disorders compared to controls, with mean increases of 0.58 in Parkinson’s disease (PD), 0.59 in AD, and 0.49 in PSP (Mohammadi et al., 2024; Muñoz-Delgado et al., 2024). Importantly, no studies have specifically evaluated peripheral immune markers in patients with both neurodegeneration and ventriculomegaly, leaving a critical gap in the field. Emerging evidence suggests a pivotal role for neuroinflammation in the pathophysiology of both ventriculomegaly and neurodegenerative disorders (Wang et al., 2020).

The current criteria for iNPH rely on a combination of clinical features, neuroimaging findings, cerebrospinal fluid (CSF) pressure measurements, and the patient’s response to CSF tap tests or shunt surgery. Key imaging biomarkers include an Evans’ index (EI) > 0.3, disproportionately enlarged subarachnoid space hydrocephalus (DESH), and a callosal angle (CA) < 90°. Clinical guidelines have identified DESH as a true prognostic marker for iNPH (Lee et al., 2023; Nakajima et al., 2021). In addition, the CA is particularly useful as it serves as an indirect indicator of DESH, aiding in the differentiation of iNPH from other conditions such as AD and normal aging (Nakajima et al., 2021). In keeping with this, recent studies highlight PSP as the neurodegenerative disorder most likely to mimic iNPH both clinically and radiologically, underscoring the importance of specific imaging features for accurate diagnosis of neurodegenerative diseases associated with ventriculomegaly (Fu et al., 2023).

However, accumulating evidence indicates that imaging results in the context of neurodegeneration are highly variable and far from pathognomonic. Features once thought to be distinctive of iNPH are increasingly recognized across certain neurodegenerative conditions, particularly PSP and LBD, demonstrating a substantial overlap in radiological phenotypes (Kawazoe et al., 2025). This convergence of imaging features complicates clinical interpretation and reinforces the need for integrative diagnostic approaches that combine imaging with biomarkers and longitudinal clinical assessments.

Despite advances in understanding individual biomarkers, correlations between brain MRI parameters and NLR have not been systematically examined in patients with ventriculomegaly and neurodegenerative disorders. Most previous studies have focused on iNPH or individual markers in isolation, leaving a critical gap regarding how imaging features relate to systemic inflammatory alterations across different neurodegenerative conditions in the context of ventriculomegaly.

Therefore, the objective of this study was to investigate peripheral immune changes and imaging features in patients with ventriculomegaly and gait impairment and to determine how these parameters differ between patients classified as neurodegenerative or probable iNPH after longitudinal follow-up.

## Materials and methods

### Study population

We retrospectively analyzed 55 patients who presented with gait impairment as the primary clinical feature, with symptom onset occurring 1.0 to 1.6 years prior to the initial consultation. At that time, they did not meet the clinical criteria for a specific neurological condition (Table 1). The study was conducted at the Movement Disorders Unit of Clínica Universidad de Navarra, Pamplona, Spain. Clinical and demographic data collected from patients between January 2019 and December 2024 were analyzed. All data were anonymized to protect patient confidentiality.

**Table 1:** Baseline demographic, clinical, and disease history characteristics of ND, iNPH, and HC groups.

Characteristics		ND (*n* = 35)	iNPH (*n* = 20)	HC (*n* = 40)	*p*-value
Gender	Men, *n* (%)	15 (43)	7 (35)	22 (55)	0.206
	Women, *n* (%)	20 (57)	13 (65)	18 (45)	
Age, years		73.9 ± 6.5	74.3 ± 5.1	74.5 ± 4.6	0.939
Symptoms duration at first visit and imaging acquisition, years		1.2 ± 0.3	1.0 ± 0.2	–	0.886
Time to diagnosis, years		3.0 ± 0.4	3.1 ± 0.2	–	0.135
Duration of disease at the end point, years		4.9 ± 0.5	5.2 ± 0.6		0.418
Parkinsonism, *n* (%)		27 (77)	13 (65)	–	
Oculomotor abnormalities (supranuclear vertical gaze palsy/impairment in vertical saccades), *n* (%)		24 (69)	–	–	
Hypophonia, *n* (%)		10 (29)	–	–	
Falls, *n* (%)		28 (80)	–	–	
Dysphagia, *n* (%)		10 (29)	–	–	

Values are means ± SD unless otherwise stated. AD, Alzheimer’s disease; LBD, Lewy body dementia; HC, healthy control; iNPH, idiopathic normal pressure hydrocephalus; ND, neurodegeneration; PD, Parkinson’s disease; PSP, progressive supranuclear palsy; SD, standard deviation.

Included subjects were patients aged 60 years or older with a Mini-Mental State Examination (MMSE) score ≥ 24, ensuring the evaluation of individuals without severe cognitive dysfunction. In addition, those with pyramidal, cerebellar, and neuromuscular dysfunction, rheumatologic disorders, or any other condition resulting in gait impairment were excluded to minimize the impact of confounding neurological deficits.

Exclusion criteria were applied prior to patient inclusion and included acute or chronic inflammatory or infectious diseases, including confirmed SARS-CoV-2 infection, as well as the use of immunosuppressive or immunomodulatory therapies or any other conditions potentially affecting peripheral blood counts. Furthermore, individuals with severe vascular encephalopathy (Fazekas Scale score ≥ 3) were also excluded (Fazekas et al., 1987). No patients were receiving dopaminergic therapy at the time of the initial consultation, as confirmed by a thorough review of their medical histories.

At baseline, patients underwent brain MRI and provided blood samples for peripheral blood cell count analysis. During the same visit, scores of the iNPH Grading Scale (iNPHGS), which assesses gait, cognitive, and urinary dysfunction, as well as the MMSE, were obtained (Kubo et al., 2008; Tombaugh and McIntyre, 1992).

After this initial evaluation, patients were followed clinically every 6 months as part of routine clinical practice, without additional research-driven investigations. Additional investigations, such as [18F]-fluorodopa ([18F]-FDOPA) PET/CT or CSF biomarkers, were requested selectively based on clinical findings suggestive of a neurodegenerative process, rather than being performed systematically in all patients. At the 5-year time point, based on longitudinal clinical assessments and supportive tests, patients were classified into neurodegenerative (ND) or iNPH groups. This final classification was independently performed by two movement disorders specialists (MRL and IAO) and was considered the ground truth, representing the diagnostic gold standard for assigning patients into the ND or iNPH groups.

All patients underwent brain MRI at the initial consultation, within a timeframe of 1.0 to 1.6 years from clinical onset (Table 1). The brain MRI acquisition protocol is detailed in the imaging subsection. Imaging-based diagnostic criteria for iNPH, specifically the presence of an EI > 0.3, CA < 90°, and features consistent with DESH, were considered imaging inclusion criteria (Nakajima et al., 2021). A total of 25 patients were excluded due to either not meeting imaging criteria or having non-evaluable sequences caused by motion artifacts; these exclusions were performed after initial clinical selection.

Additionally, a healthy control (HC) group of 40 age-matched participants was included. Blood cell counts and brain MRI parameters were analyzed in these subjects for comparative evaluation at baseline. The imaging data and blood cell count results for the control group were obtained from the Neurology and Radiology Departments’ database at our hospital.

### Blood cell count

Fasting blood samples were collected from an antecubital vein in the morning on the same day as the initial consultation. Patients were not receiving medications that could potentially affect hematologic cell counts. An automated hematology analyzer was used for leukocyte counts, including neutrophils, lymphocytes, monocytes, eosinophils, and basophils. The NLR was calculated for all patients (Zahorec, 2001).

### Imaging

Imaging was performed on a 3 T MRI scanner (MAGNETOM® Skyra, Siemens Healthineers AG, Forchheim, Germany) using a 32-channel head coil. Brain MRI protocol included a T1-weighted anatomical 3D magnetization prepared rapid gradient echo imaging (MPRAGE) sequence with 1-mm isotropic resolution, and a T2-weighted fluid attenuated inversion recovery (FLAIR) sequence was included.

All the imaging parameters were measured by the same radiologist (M. J. V), who was blinded to the patient’s clinical features.

**Figure 1:**
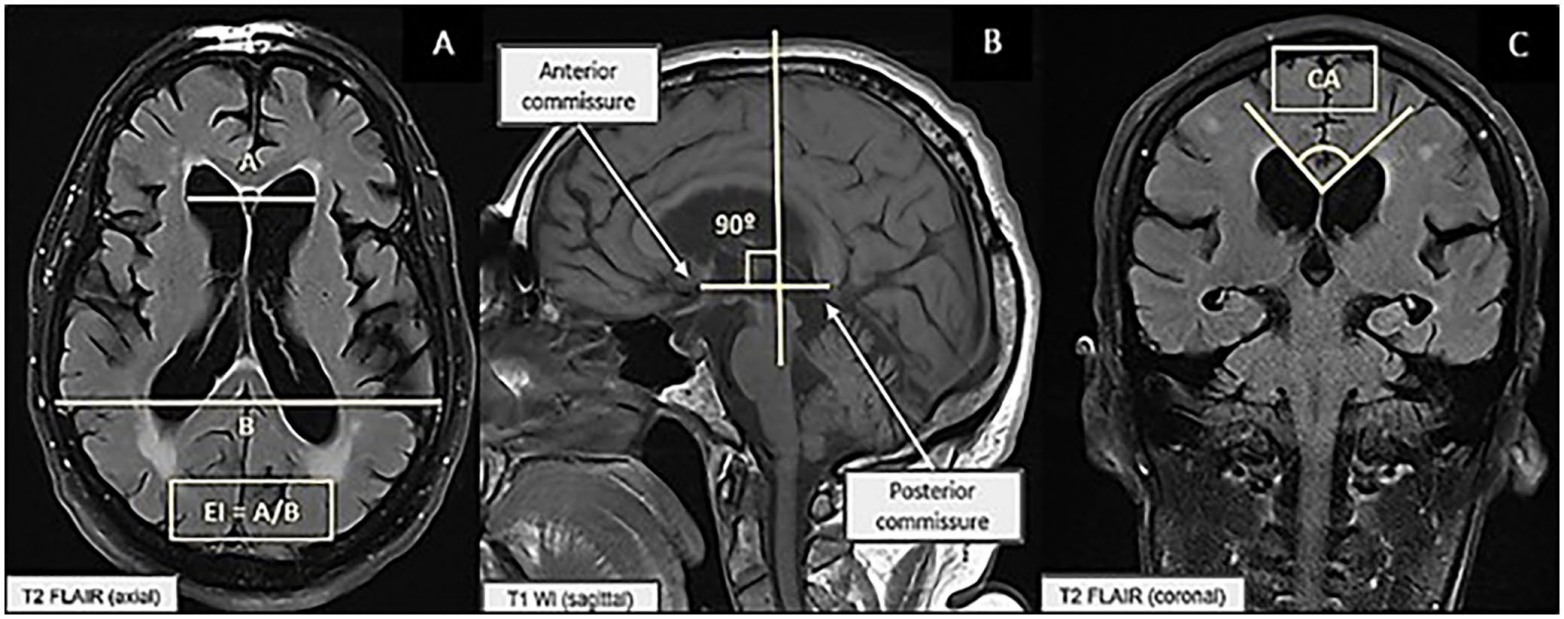
**(A)** Measurement of the Evans index. **(B)** Measurement of the callosal angle on sagittal T2 FLAIR (Fluid-Attenuated Inversion Recovery) brain MRI. **(C)** Measurement of the callosal angle on coronal T1-weighted brain MRI.

The EI was calculated in the axial plane by measuring the maximum width of the frontal horns divided by the maximum width of the inner skull in the horizontal plane, measured at the level of the III ventricle, with values > 0.3 indicating ventriculomegaly (Nakajima et al., 2021) (Figure 1A). The CA was calculated in the coronal plane, perpendicular to the anterior commissure–posterior commissure line, at the level of the posterior commissure and between the medial walls of the lateral ventricles, with a steep angle defined as < 90° (Nakajima et al., 2021) (Figures 1B,C).

The DESH score comprised five items: ventriculomegaly, dilated Sylvian fissures, tight high convexity, CA, and focal sulcal dilation. Each item was rated from 0 to 2, for a maximum total score of 10 (Nakajima et al., 2021). The CA was calculated independently of the DESH score.

### Lumbar puncture

To evaluate the relevance of ventriculomegaly in the development of clinical features, all patients underwent a standardized lumbar tap test at a mean of 1.30 years [standard deviation (SD) = 0.3] after symptom onset, performed under sterile conditions (Wikkelsö et al., 1986). In each case, the opening pressure was measured, and controlled removal of 50 mL of CSF was performed. Routine CSF analysis, including cell count, total protein, and glucose levels, was obtained. Clinical response was evaluated within 24 h using the Timed Up and Go (TUG) test and patients’ subjective clinical reports (Sundström et al., 2022). A positive response was defined as either objective gait improvement on this test or subjective motor benefit reported by the patient within 24 h after the lumbar puncture. CSF biomarkers, including Aβ42, total tau (t-tau), and phosphorylated tau (p-tau), were evaluated in patients who exhibited marked cognitive impairment suggestive of AD, based on clinical observations documented during follow-up.

### Group classification

Patients were classified into two groups based on longitudinal clinical profiles and supporting data reviewed at the 5-year timepoint assessment. The first group consisted of individuals who, at their last clinical assessment (M = 4.9 years, SD = 0.5), fulfilled established criteria for a defined neurodegenerative condition, such as PSP, AD, LBD, and PD, according to the presence of MDS-PSP criteria (Höglinger et al., 2017), NIA-AA Research Framework (Jack et al., 2018), LBD consortium criteria (McKeith et al., 2017), and MSD-PD criteria (Postuma et al., 2015), respectively. Patients whose clinical evolution remained consistent with these diagnoses were assigned to the neurodegenerative (ND) group. CSF biomarkers, including Aβ42, t-tau, and p-tau, along with cognitive assessments and [^18^F]-FDOPA PET/CT, were used as complementary tools to support the diagnosis of specific neurodegenerative conditions.

The second group comprised patients who, at the time of the last assessment (*M* = 5.2 years, SD = 0.6), fulfilled current criteria for probable iNPH, based on brain MRI measurements including EI, CA, and DESH, clinical manifestations, CSF opening pressure, and response to the tap test (Nakajima et al., 2021).

### Statistical analysis

Quantitative variables were summarized with the mean and standard deviation (SD), and frequencies were calculated for categorical variables. The normality assumption was checked using the Shapiro–Wilk test. Group comparisons among patients were performed using Pearson’s chi-squared test or Fisher’s exact test for categorical variables. One-way ANOVA, the *t*-test, Kruskal–Wallis test, and Mann–Whitney *U*-test were used for comparing quantitative variables. The Bonferroni correction was applied to adjust for multiple comparisons. The effect size (ES) and 95% confidence interval were calculated using Cohen’s d method. The area under the receiver operating characteristic curve (AUC) and 95% confidence interval (95% CI) were estimated to quantify the capacity of the clinical and brain MRI scales performed at baseline to discriminate between ND and iNPH groups. The optimal cutoff points, including those used to evaluate the association between NLR and tap test response, were estimated using the Youden Index. Spearman’s rank correlation coefficients (r_s_) were used to explore correlations between variables. A two-sided *p*-value of < 0.05 was considered statistically significant. All statistical analyses were conducted with Stata (StataCorp. 2023. Stata Statistical Software: Release 18. College Station, TX: StataCorp LLC).

## Results

### Demographic and baseline clinical features

Table 1 summarizes the demographic features of the ND, iNPH, and HC groups. The age distribution was comparable across all groups.

At the first assessment, the reported onset of clinical manifestations was *M* = 1.2 years, SD = 0.3 in the ND group and *M* = 1.0 years, SD = 0.2 in the iNPH group. The MMSE score was *M* = 26.4, SD = 1.8 in the ND group and M = 27.7, SD = 2.2 in the iNPH group.

Baseline assessment showed significantly higher scores on the iNPHGS, including the cognitive, urinary, and gait subcategories, and the total score, in the ND group compared to the iNPH group (*M* = 6.5, SD = 1.7 vs. *M* = 5.1, SD = 0.6, *p* < 0.001; ES = 1.1, 95% CI [0.5, 1.7]) (Table 2). The AUC was 0.81 at the established cutoff value of 6. At this threshold, the scale demonstrated a sensitivity of 74% and a specificity of 80% in distinguishing patients with ND from those with iNPH (Table 3).

**Table 2:** Clinical severity, cognitive assessment, peripheral immune pattern, and imaging features according to ND, iNPH, and HC groups.

Characteristics	ND (*n* = 35)	iNPH (*n* = 20)	HC (*n* = 40)	*p*-value
Clinical severity				
iNPHGS, cognitive score	2.2 ± 0.5	1.9 ± 0.5	–	0.028
iNPHGS, gait subscore	2.1 ± 0.8	1.7 ± 0.5	–	0.023
iNPHGS, urinary subscore	2.3 ± 0.9	1.6 ± 0.5	–	<0.001
iNPHGS, total score	6.5 ± 1.7	5.1 ± 0.6	–	<0.001
Cognitive assessment				
MCI amnesic, *n* (%)	11 (31)	7 (35)	–	0.010
MCI non-amnesic, *n* (%)	18 (51)	3 (15)	–	
Normal cognition, *n* (%)	6 (17)	10 (50)	–	
MMSE	26.4 ± 1.8	27.7 ± 2.2	–	0.024
Immunological parameters				
WBC, 1000/mL	7.5 ± 1.3	7.1 ± 1.4	6.8 ± 0.8	0.039
Neutrophils, 1,000/mL	60.7 ± 6.7	57.0 ± 7.7	55.6 ± 2.8	<0.001
Lymphocytes, 1,000/mL	26.6 ± 4.7	30.5 ± 4.8	34.8 ± 2.3	<0.001
NLR	2.4 ± 0.5	1.9 ± 0.4	1.6 ± 0.2	<0.001
Imaging features				
Evans’ index	0.33 ± 0.02	0.35 ± 0.02	0.27 ± 0.01	<0.001
Callosal angle	80.0 ± 5.3	87.5 ± 2.0	129.7 ± 3.5	<0.001
DESH score	6.4 ± 1.4	5.0 ± 1.1	0.1 ± 0.3.	<0.001

Values are means ± SD unless otherwise stated. Post hoc analyses for all pairwise comparisons were performed with Bonferroni correction to account for multiple testing. All comparisons remained statistically significant after adjustment. Detailed *p*-values are provided in Supplementary Table 1. AD, Alzheimer’s disease; DESH score, disproportionately enlarged subarachnoid space hydrocephalus score; LBD, Lewy body dementia; HC, healthy control; iNPH, idiopathic normal pressure hydrocephalus; iNPHGS, iNPH Grading Scale; MCI, Mild Cognitive Impairment; MMSE, Mini-Mental State Examination; ND, neurodegeneration; NLR, neutrophil-to-lymphocyte ratio; PD, Parkinson’s disease; PSP, progressive supranuclear palsy; SD, standard deviation; WBC, white blood cell count.

**Table 3:** AUC values for NLR, iNPHGS, EI, CA, and DESH in differentiating ND from the iNPH group.

Measure	AUC (95% CI)	Cutoff value	Sensitivity	Specificity
iNPHGS	0.81 (0.70–0.91)	6	74%	80%
NLR	0.79 (0.66–0.92)	2.0	80%	70%
EI	0.78 (0.66–0.90)	0.34	85%	63%
CA	0.92 (0.85–0.99)	84	95%	75%
DESH	0.78 (0.66–0.91)	6	80%	70%

95% CI, confidence interval; AUC, area under the receiving operating curve; CA, callosal angle; DESH score, disproportionately enlarged subarachnoid space hydrocephalus score; EI, Evan’s index; iNPHGS, iNPH grading scale; NLR, neutrophil-to-lymphocyte ratio. The ND group comprised patients with PSP, AD, LBD, and PD diagnosed according to established criteria (Höglinger et al., 2017; Jack et al., 2018; McKeith et al., 2017; Postuma et al., 2015). The iNPH group was defined following current criteria (Nakajima et al., 2021). All final clinical diagnoses were independently confirmed by two movement disorders specialists (MRL and IAO) and served as the reference standard for subsequent analyses.

### Immune parameters

At baseline, the NLR was significantly different among groups (*p* < 0.001). The ND group showed a higher NLR (M = 2.4, SD = 0.5) than both the iNPH group (*M* = 1.9, SD = 0.4, *p* < 0.001; ES = 1.0, 95% CI [0.5, 1.6]) and HC (*M* = 1.6, SD = 0.2, *p* < 0.001; ES = 2.1, 95% CI [1.6, 2.7]) (Table 2). The AUC to discriminate between ND and iNPH groups was 0.79; the cutoff value of 2.0 yielded a sensitivity of 80% and a specificity of 70% (Table 3).

### Imaging features

Baseline MRI measurements (EI, CA, and DESH score) differed significantly among groups (*p* < 0.001). The iNPH group exhibited a higher EI (*M* = 0.35, SD = 0.02) compared to both the ND group (M = 0.33, SD = 0.02, *p* < 0.001; ES = 0.8, 95% CI [0.3, 1.4]) and HC (*M* = 0.27, SD = 0.01, *p* < 0.001; ES = 3.8, 95% CI [3.0, 4.6]). Notably, the CA was significantly lower in the ND group (*M* = 80.0, SD = 5.3) compared to the iNPH group (*M* = 87.5, SD = 2.0, *p* < 0.001; ES = 1.7, 95% CI [1.1, 2.3]) and HC (*M* = 129.7, SD = 3.5, *p* < 0.001; ES = 11.2, 95% CI [9.3, 13.1]) (Table 2).

Additionally, the DESH score was significantly higher in the ND group (*M* = 6.4, SD = 1.4) than in the iNPH group (*M* = 5.0, SD = 1.1, *p* < 0.001; ES = 1.1, 95% CI [0.5, 1.6]). Both patient groups showed elevated values relative to HC (M = 0.10, SD = 0.3; *p* < 0.001; ES for the ND group = 6.4, 95% CI [5.3, 7.5]; ES for the iNPH group = 7.1, 95% CI [5.7, 8.5]) (Table 2).

The diagnostic performance of neuroimaging markers in distinguishing ND from iNPH is summarized in Table 3. EI showed an AUC of 0.78, with 85% sensitivity and 63% specificity at a cutoff of 0.34. CA achieved the highest accuracy (AUC = 0.92), with 95% sensitivity and 75% specificity at a cutoff of 84. DESH yielded an AUC of 0.78, with a sensitivity of 80% and specificity of 70% at a cutoff of 6.

### Tap test response

Of the 55 patients who underwent the tap test, 62% (*n* = 34) showed a positive response. At the timepoint assessment, 47% of these responders (*n* = 16) were included in the ND group, including PSP (*n* = 10), AD (*n* = 4), LBD (*n* = 1), and PD (*n* = 1), while the remaining 53% (*n* = 18) were classified as probable iNPH. Patients with clinical improvement were referred to neurosurgical evaluation. Of those referred, 18 maintained a sustained shunting response at 36 months and were ultimately classified as probable iNPH. Among the 21 non-responders, 90% (*n* = 19) were ultimately diagnosed with a neurodegenerative disorder, and 10% (*n* = 2) continued to meet criteria for probable iNPH.

### Clinical features during follow-up

During the course of the disease, 71% of patients exhibited parkinsonism; 44% had oculomotor abnormalities such as supranuclear vertical gaze palsy or impaired vertical saccades, 19% presented hypophonia, 19% exhibited dysphagia, and 50% experienced falls. Detailed clinical characteristics for each group are presented in Table 1.

### Cognitive assessment

All patients underwent cognitive assessment during the course of the disease. Within the ND group, 51% had non-amnesic mild cognitive impairment (naMCI), 31% had amnesic MCI (aMCI), and 17% exhibited a normal cognitive profile. In contrast, the iNPH group showed 35% with aMCI, 15% with naMCI, and 50% with a normal cognitive profile. Detailed cognitive profiles are summarized in Table 2.

### Additional diagnostic studies

Guided by clinical findings recorded throughout the course of the disease, CSF biomarkers were analyzed in 25% (*n* = 14) of the cohort to further investigate cognitive decline suggestive of AD. Among these patients, 57% (*n* = 8) exhibited biomarker profiles consistent with AD, with *M* = 483.4 pg./mL, SD = 174.0 for Aβ42, *M* = 283.4 pg./mL, SD = 62.9 for total tau, and *M* = 29.9 pg./mL, SD = 11.9 for phosphorylated tau. The remaining 43% (*n* = 6), who did not show abnormal CSF biomarker levels at the last assessment, were classified within the iNPH group.

Furthermore, [^18^F]-FDOPA PET/CT imaging was performed in 71% of patients presenting with ventriculomegaly and parkinsonism to evaluate dopaminergic function and assist in differential diagnosis among PD, PSP, and LBD. Patients were drug-naïve or had discontinued dopaminergic treatment at least 12 h before imaging. A significant striatal [^18^F]-FDOPA PET/CT uptake decrease was observed in 72% (*n* = 28) of patients. Among those with reduced uptake, 75% (*n* = 21) were diagnosed with PSP, 14% (*n* = 4) with iNPH, 7% (*n* = 2) with LBD, and 4% (*n* = 1) with PD. The remaining 28% (*n* = 11) demonstrated normal striatal uptake.

### Final diagnosis

At the 5-year timepoint assessment, a comprehensive review of clinical data revealed that 44% (*n* = 24) of patients with ventriculomegaly met clinical criteria for PSP, 14% (*n* = 8) for AD, 4% (*n* = 2) for LBD, and 2% (*n* = 1) for PD. The remaining 36% (*n* = 20) fulfilled the criteria for probable iNPH.

Among the 24 patients with a final diagnosis of PSP, the most common phenotype was PSP with predominant freezing of gait (PSP-FOG) observed in 63% (*n* = 15) of cases, followed by Richardson syndrome (PSP-RS) in 29% (*n* = 7). The parkinsonian (PSP-P) and corticobasal syndrome (PSP-CBS) variants were less frequent, each accounting for 4% (*n* = 1). Notably, 92% (*n* = 22) of PSP patients exhibited no response to levodopa in the follow-up, while 8% (*n* = 2) experienced only a transient benefit lasting less than 1 year.

The time to clinical diagnosis, estimated from longitudinal clinical evaluations and medical record review, was comparable between groups, with no significant differences found, averaging *M* = 3.0 years, SD = 0.4 for the ND group and *M* = 3.1 years, SD = 0.2 for the iNPH group (Table 1). Patients were followed for up to 5 years, which allowed confirmation of the final diagnosis used for classification.

### Correlations and associations between clinical severity, NLR, neuroimaging parameters, tap test response, and diagnosis

A significant positive correlation was observed between baseline NLR and total iNPHGS in the ND group (*r_s_* = 0.48, *p* = 0.004). In contrast, no significant correlation between these parameters was found in the iNPH group (*r*_s_ = 0.08, *p* = 0.731).

Additionally, NLR showed no correlation with the imaging parameters analyzed, including EI, CA, and DESH, in either the ND or iNPH groups. No associations were observed between immune parameters and these imaging measures.

A significant association was found between NLR levels and tap test response, which differed by group. In the ND group, higher NLR (>2.4) was strongly associated with a lack of response, with 94% of non-responders exceeding this threshold. Conversely, in the iNPH group, lower NLR (<2.2) was linked to a positive response, as all patients (100%) below this cutoff showed improvement (*p* < 0.001).

## Discussion

Our study demonstrates that patients with gait impairment and ventriculomegaly exhibit differences in clinical severity, imaging markers, and immune response, which may aid in differentiating neurodegenerative disorders from iNPH. Compared with iNPH subjects, the ND group had higher iNPHGS scores, elevated NLR, lower CA, and higher DESH scores. ROC analyses highlighted the discriminative power of NLR (with 80% sensitivity, 70% specificity) and CA (with 95% sensitivity, 86% specificity). Notably, within the ND group, baseline NLR levels showed a significant positive correlation with the iNPHGS score, suggesting a potential link between systemic inflammation and clinical severity. NLR levels were also significantly associated with tap test response, showing opposite patterns, with higher NLR levels linked to non-response in the ND group, whereas lower NLR levels were consistently associated with improvement in probable iNPH.

Numerous studies have highlighted the frequent coexistence of iNPH with neurodegenerative disorders (Espay et al., 2017). Among them, AD is a well-recognized comorbidity, in which impaired CSF reabsorption could potentially be caused by brain amyloid deposition, leading to ventricular enlargement (Cagnin et al., 2015). Consistent with this, 23% of the patients in our cohort met AD criteria at the final evaluation. On the other hand, PSP is now recognized as the most common neurodegenerative condition mimicking iNPH, particularly in patients with late-onset vertical gaze palsy (Ohara et al., 2020; Fu et al., 2023). Data from the Queen Square Brain Bank and the University of Cincinnati reinforce this coexistence (Magdalinou et al., 2013). In our study, PSP was the most common diagnosis within the ND group, with the progressive freezing-of-gait PSP variant being the most prevalent, likely attributable to our gait-focused inclusion criteria. The marked subcortical atrophy with minimal cortical involvement described in this PSP variant may explain ventricular enlargement (Kovacs et al., 2020; Pavone et al., 2023). Although LBD has been linked to ventriculomegaly, the limited number of LBD patients in our cohort prevented further association analyses (Espay et al., 2017).

Building on clinical characteristics, a notable proportion of iNPH patients exhibited parkinsonism (Mostile et al., 2022). Nevertheless, decreased [^18^F]-FDOPA striatal uptake was observed in only 14% of these patients, compared with 62% in the ND group, later diagnosed with PSP, LBD, or PD. Although dopaminergic denervation in iNPH has been reported, its clinical significance remains uncertain (Mostile et al., 2022). This may result either from a concurrent neurodegenerative process or from mechanical disruption of the nigrostriatal pathway caused by ventricular enlargement (Palermo et al., 2025). This is supported by the symmetric caudate nucleus [^18^F]-FDOPA uptake reduction in iNPH, in contrast to the asymmetric rostrocaudal putamen reduction typical of PD or the diffuse striatal involvement in other parkinsonian syndromes (Pozzi et al., 2021; Nicastro et al., 2023). Reduced postsynaptic D2 receptor density in iNPH further supports this notion (Ouchi et al., 2007).

In our cohort, the ND group exhibited the highest baseline NLR levels compared with both the iNPH group and healthy controls, indicating a shift toward a proinflammatory state. Consistent with prior findings in patients with neurodegenerative disorders, PSP patients in our cohort exhibited elevated NLR regardless of ventricular size, suggesting that neurodegeneration, rather than ventriculomegaly, may underlie systemic inflammation in this group (Inci, 2020; Muñoz-Delgado et al., 2023). It is important to note that NLR values can be influenced by neuroendocrine factors, including norepinephrine/epinephrine, which affect lymphocyte counts, and cortisol, which affects neutrophils. Dysregulation of these systems has been described in neurodegenerative diseases and iNPH, potentially contributing to the correlations observed in our cohort (Beis et al., 2018; Vida et al., 2024). The significant correlation between baseline NLR and iNPHGS within the ND group supports the role of peripheral immune factors in disease severity, despite the absence of longitudinal data.

Baseline NLR was associated with tap test outcomes. Higher NLR predicted non-response in the ND group, particularly among PSP patients with ventriculomegaly, whereas lower NLR was linked to a favorable response in iNPH. These results add clinical relevance to NLR as a potential early biomarker of neurodegeneration and treatment responsiveness (Bissacco et al., 2023; Uchigami et al., 2025).

Neuroimaging markers such as EI, CA, and DESH remain essential in iNPH diagnosis, with EI useful for initial screening. However, overlapping features with PSP and LBD complicate differentiation (Ohara et al., 2020; Kawazoe et al., 2025). In accordance with prior reports, significant differences in EI were found between ND and iNPH patients in our cohort (Kawazoe et al., 2025). CA was the most discriminative marker, with lower values in ND, consistent with associations seen in PSP and PD. Higher CA values reported in AD were not observed, likely due to the low AD representation and gait-focused inclusion criteria (Nakajima et al., 2021; Cagnin et al., 2015; Ohara et al., 2020). Notably, DESH scores were higher in the ND group, suggesting that this pattern is not exclusive to iNPH and may reflect broader glymphatic or CSF dynamic alterations seen across several disorders (Kawazoe et al., 2025; Petersen et al., 2024). Although DESH has been considered a marker for iNPH and a predictor of shunt response, its presence in neurodegenerative diseases calls for cautious interpretation alongside clinical data to improve diagnostic accuracy and avoid misclassification (Fu et al., 2023; Jóhannsdóttir et al., 2024; Rovira and Hodel, 2022).

Given the subtle onset and non-specific symptoms of iNPH, integrating iNPHGS, blood biomarkers, cognitive testing, imaging, and tap test results improves differential diagnosis and treatment planning. Notably, over half of patients with gait disturbances and ventriculomegaly were ultimately diagnosed with neurodegenerative disorders, underscoring the need for longitudinal monitoring.

Despite the strengths of this study, several limitations should be acknowledged. The retrospective design restricts the ability to draw causal inferences and may introduce selection bias. The modest sample size limited the control for potential confounders such as age and disease duration. NLR values may be influenced by neuroendocrine factors, such as norepinephrine/epinephrine, which affect lymphocyte counts, and cortisol, which affects neutrophils. To minimize this potential bias, all blood samples in our study were collected in the early morning under fasting conditions; however, residual effects cannot be completely excluded and should be considered when interpreting our results. Furthermore, while our findings support NLR as a potential biomarker, the modest sample size limited the exploration of combining multiple significant parameters to enhance diagnostic accuracy. The correlations identified in our study were of moderate strength. This indicates that, although statistically significant, they should be interpreted with caution and regarded as supportive rather than definitive evidence, thereby enhancing transparency and interpretability. Variability in CA measurements due to differences in slice selection and patient positioning highlights the need for standardized imaging protocols. Additionally, all imaging assessments were performed by a single radiologist, preventing evaluation of inter-rater reliability and potentially limiting reproducibility. The ND group was heterogeneous and predominantly composed of PSP cases, which may limit generalizability. A subanalysis of the PSP subgroup yielded consistent results (see Supplementary Tables 2, 3); however, the specificity of these biomarkers to PSP vs. other neurodegenerative disorders remains unclear. Taken together, these limitations emphasize the need for larger and more diverse cohorts to validate our findings and to investigate whether integrating NLR with clinical scales and imaging biomarkers could improve the differential diagnosis between neurodegenerative diseases and iNPH.

In conclusion, our findings underscore the potential of NLR as a valuable biomarker to support the differential diagnosis between neurodegenerative conditions and iNPH, reflecting systemic inflammation associated with disease severity. Given the frequent overlap in imaging characteristics between these groups, reliance on a single parameter may be misleading. A multimodal approach integrating immune, clinical, and imaging biomarkers appears to be the most appropriate strategy to enhance diagnostic accuracy and guide therapeutic decision-making.

## Data Availability

The original contributions presented in the study are included in the article/Supplementary material, further inquiries can be directed to the corresponding author/s.

## Glossary

AD: Alzheimer’s disease
AUC: Area under the receiver operating characteristic curve
CA: Callosal angle
DESH: Disproportionately enlarged subarachnoid space hydrocephalus
EI: Evans’ index
FDOPA: [^18^F] fluorodopa
HC: Healthy controls
iNPH: Idiopathic normal pressure hydrocephalus
iNPHGS: Idiopathic normal pressure hydrocephalus grading scale
LBD: Lewy body dementia
M: Mean
MMSE: Mini-Mental State Examination
NLR: Neutrophil-to-lymphocyte ratio
ND: Neurodegenerative disorder
PD: Parkinson’s disease
PET/CT: Positron emission tomography/computed tomography
PSP: Progressive supranuclear palsy
TUG: Timed Up and Go test
t tau: Total tau
p tau: Phosphorylated tau
naMCI: Non-amnesic mild cognitive impairment
aMCI: Amnesic mild cognitive impairment
ES: Effect size
