# Supplementary material for: Peripheral immunity patterns, imaging features, and clinical outcomes in patients with gait impairment and ventriculomegaly on brain MRI
Source: Front Aging Neurosci. 2025 Oct 20;17:1685288. doi: 10.3389/fnagi.2025.1685288 (PMC12580214; doi:10.3389/fnagi.2025.1685288)
Supplement: Supplementary file 1 [file Table_1.docx]

**Supplementary Material**

**Supplementary Table. 1 Post hoc pairwise comparisons of clinical and imaging variables between ND, iNPH, and HC groups**

| **Variable** | **Group 1** | **Group 2** | **Post hoc p value** |
| --- | --- | --- | --- |
| NLR | ND | iNPH | < 0.001 |
| NLR | ND | HC | < 0.001 |
| NLR | iNPH | HC | 0.002 |
| EI | ND | iNPH | < 0.001 |
| EI | ND | HC | < 0.001 |
| EI | iNPH | HC | 0.002 |
| CA | ND | iNPH | < 0.001 |
| CA | ND | HC | < 0.001 |
| CA | iNPH | HC | < 0.001 |
| DESH | ND | iNPH | < 0.001 |
| DESH | ND | HC | < 0.001 |
| DESH | iNPH | HC | < 0.001 |

**Note:** Post hoc pairwise p values for comparisons between ND, iNPH, and HC groups are shown. Statistical significance was assessed using appropriate multiple comparison corrections. **Abbreviations:** CA, Callosal angle; DESH score, Disproportionately Enlarged Subarachnoid Space Hydrocephalus score; EI, Evan’s index; HC, Healthy controls; iNPH, Idiopathic Normal Pressure Hydrocephalus; ND, Neurodegeneration; NLR, Neutrophil-to-lymphocyte ratio; PD, Parkinson’s Disease; PSP, Progressive Supranuclear Palsy.

**Supplementary Table 2. Demographic features and study results in PSP, iNPH, and HC groups.**

|  | | **PSP (*n*=24)** | **iNPH (*n*=20)** | **HC (*n*=40)** | ***p* value** |
| --- | --- | --- | --- | --- | --- |
| Sex | Men, *n* (%) | 11 (46) | 7 (35) | 22 (55) | 0.244 |
|  | Women, *n* (%) | 13 (53) | 13 (65) | 18 (45) |  |
| Age, years | | 74.1 ± 4.4 | 74.3 ± 5.1 | 74.5 ± 4.6 | 0.927 |
| Clinical severity | | | | | |
| iNPHGS, cognitive score | | 2.1 ± 0.5 | 1.9 ± 0.5 | - | 0.149 |
| iNPHGS, gait subscore | | 1.9 ± 0.7 | 1.7 ± 0.5 | - | 0.223 |
| iNPHGS, urinary subscore | | 1.9 ± 0.5 | 1.6 ± 0.5 | - | 0.047 |
| iNPHGS, total score | | 5.9 ± 1.0 | 5.1 ± 0.6 | - | g0.002 |
| Cognitive assessment | | | | | |
| MCI amnesic, *n* (%) | | 2 (8) | 7 (35) | - | 0.001 |
| MCI non-amnesic, *n* (%) | | 16 (67) | 3 (15) | - |  |
| Normal cognition, *n* (%) | | 6 (25) | 10 (50) | - |  |
| MMSE | | 26.5 ± 2.0 | 27.7 ± 2.2 | - | 0.001 |
| Immunological parameters | | | | | |
| WBC, 1000/mL | | 7.5 ± 1.2 | 7.1 ± 1.4 | 6.8 ± 0.8 | 0.039 |
| Neutrophils, 1000/mL | | 61.6 ± 6.7 | 57.0 ± 7.7 | 55.6 ± 2.8 | < 0.001 |
| Lymphocytes, 1000/mL | | 26.5 ± 4.3 | 30.5 ± 4.8 | 34.8 ± 2.3 | < 0.001 |
| NLR | | 2.4 ± 0.5 | 1.9 ± 0.4 | 1.6 ± 0.2 | < 0.001 |
| Imaging features | | | | | |
| Evans’ index | | 0.33 ± 0.02 | 0.35 ± 0.02 | 0.27 ± 0.01 | < 0.001 |
| Callosal angle | | 79.9 ± 5.8 | 87.5 ± 2.0 | 129.7 ± 3.5 | < 0.001 |
| DESH score | | 6.4 ± 1.6 | 5.0 ± 1.1 | 0.1 ± 0.3. | < 0.001 |

*Note:* Values are means ± SD unless otherwise stated. **Abbreviations:** AD, Alzheimer’s disease; DESH score, Disproportionately Enlarged Subarachnoid Space Hydrocephalus score; LBD, Lewy Body Dementia; HC, healthy control; iNPH, Idiopathic Normal Pressure Hydrocephalus; iNPHGS, iNPH Grading Scale; MCI, Mild Cognitive Impairment; MMSE, Mini-Mental State Examination; ND, Neurodegeneration; NLR, Neutrophil-to-lymphocyte ratio; PD, Parkinson’s Disease; PSP, Progressive Supranuclear Palsy; SD, standard deviation; WBC, White Blood Cell Count.

**Supplementary Table 3. AUC values for NLR, iNPHGS, EI, CA, DESH in differentiating PSP from iNPH group**

| Measure | AUC  (95% CI) | Cutoff value | Sensitivity | Specificity |
| --- | --- | --- | --- | --- |
| iNPHGS | 0.75 (0.61-0.88) | 5.5 | 67% | 80% |
| NLR | 0.79 (0.65-0.93) | 2.3 | 63% | 95% |
| EI | 0.79 (0.66-0.93) | 0.34 | 85% | 63% |
| CA | 0.90 (0.81-1.0) | 84 | 95% | 83% |
| DESH | 0.79 (0.67-0.92) | 5.5 | 75% | 70% |

**Abbreviations:** 95% CI, Confidence interval; AUC, Area under the receiving operating curve; CA, Callosal angle; DESH score, Disproportionately Enlarged Subarachnoid Space Hydrocephalus score; EI, Evan’s index; iNPHGS, iNPH Grading Scale; NLR, neutrophil-to-lymphocyte ratio, PSP, Progressive Supranuclear Palsy.
